# Ten years of hand hygiene excellence: a summary of outcomes, and a comparison of indicators, from award-winning hospitals worldwide

**DOI:** 10.1186/s13756-024-01399-0

**Published:** 2024-04-19

**Authors:** Ermira Tartari, Jacopo Garlasco, Marcela Hernández-de Mezerville, Moi Lin Ling, Hilda Márquez-Villarreal, Wing-Hong Seto, Anne Simon, Thomas-Jörg Hennig, Didier Pittet

**Affiliations:** 1 Faculty of Health Sciences, University of Malta, 2080 Msida, Malta; 2 Infection Prevention and Control Unit, Department of Integrated Health Services, WHO Headquarters, Geneva, Switzerland; 3 Infectious Diseases Unit, Department of Diagnostics and Public Health, University of Verona, Verona, Italy; 4 Hospital Nacional de Niños, San José, Costa Rica; 5 Infection Prevention and Epidemiology, Singapore General HospitalSingapore, 169608 Singapore, Singapore; 6 Department of Public Health, University of Guadalajara, Jalisco, Mexico; 7 School of Public Health, WHO Collaborating Centre, The University of Hong Kong, Hong Kong, China; 8 Infection Control and Prevention, CHU Helora, Haine-Saint-Paul, Belgium; 9 Medical Division, Borer Chemie AG, Zuchwil, Switzerland; 10 Faculty of Medicine & Clean Hospitals, University of Geneva, Geneva, Switzerland

**Keywords:** Hand hygiene, Alcohol-based handrub, World health organization, Infection prevention and control, WHO multimodal improvement strategy, WHO hand hygiene self-assessment framework, Patient safety, Global health, Awards, Excellence

## Abstract

**Background:**

Hand hygiene is a crucial measure for the prevention of healthcare-associated infections (HAIs). The Hand Hygiene Excellence Award (HHEA) is an international programme acknowledging healthcare facilities for their leadership in implementing hand hygiene improvement programmes, including the World Health Organisation’s Multimodal Improvement Strategy. This study aimed at summarising the results of the HHEA campaign between 2010 and 2021 and investigating the relationship between different hand hygiene parameters based on data from participating healthcare facilities.

**Methods:**

A retrospective analysis was performed on datasets from HHEA forms, including data on hand hygiene compliance, alcohol-based handrub (ABHR) consumption, and Hand Hygiene Self-Assessment Framework (HHSAF) scores. Descriptive statistics were reported for each variable. The correlation between variables was inspected through Kendall’s test, while possible non-linear relationships between hand hygiene compliance, ABHR consumption and HHSAF scores were sought through the Locally Estimated Scatterplot Smoothing or logistic regression models. A tree-structured partitioning model was developed to further confirm the obtained findings.

**Results:**

Ninety-seven healthcare facilities from 28 countries in three world regions (Asia-Pacific, Europe, Latin America) were awarded the HHEA and thus included in the analysis. HHSAF scores indicated an advanced hand hygiene promotion level (median 445 points, IQR 395–480). System change (100 [95–100] points) and institutional safety climate (85 [70–95] points) showed the highest and lowest score, respectively. In most cases, hand hygiene compliance was above 70%, with heterogeneity between countries. ABHR consumption above 20 millilitres per patient-day (ml/PD) was widely reported, with overall increasing trends. HHSAF scores were positively correlated with hand hygiene compliance (*τ* = 0.211, *p* = 0.007). We observed a positive correlation between compliance rates and ABHR consumption (*τ* = 0.193, *p* < 0.001), although the average predicted consumption was stable around 55–60 ml/PD for compliance rates above 80–85%. Logistic regression and partitioning tree analyses revealed that higher HHSAF scores were more likely in the high-ABHR consumption group at cut-offs around 57–59 ml/PD.

**Conclusion:**

Ten years after its inception, the HHEA proves to be a valuable hand hygiene improvement programme in healthcare facilities worldwide. Consistent results were provided by the different hand hygiene indicators and the HHSAF score represents a valuable proxy measure of hand hygiene compliance.

**Supplementary Information:**

The online version contains supplementary material available at 10.1186/s13756-024-01399-0.

## Background

The health and economic imperatives of healthcare-associated infections (HAIs) and antimicrobial resistance (AMR) are of significant global concern [1, 2]. Effective infection prevention and control (IPC) is key to prevent HAIs and reduce the transmission of multidrug-resistant pathogens in healthcare settings [3]. Hospitalised patients are vulnerable to acquiring multidrug-resistant organisms from the hands of transiently colonised healthcare workers [4]. Hand hygiene promotion is an integral, evidence-based intervention for infection prevention. Improving hand hygiene practices reduces the incidence of HAI and the spread of AMR [4–6].

The World Health Organization (WHO) has undertaken initiatives aimed at improving hand hygiene practices globally. In 2009, the WHO introduced the Multimodal Hand Hygiene Improvement Strategy (MMIS) and Implementation Toolkit, including the Hand Hygiene Self-Assessment Framework (HHSAF), to evaluate the implementation of hand hygiene promotion strategies and assess improvements over time [7, 8]. The implementation of a MMIS has been shown to be effective in increasing hand hygiene compliance and reducing HAIs [9–11]. Monitoring and evaluating hand hygiene compliance within healthcare facilities has been established globally as a key performance indicator of patient quality and safety programs [10, 12]. A global survey conducted in 2019 using the HHSAF reported that most healthcare facilities had an intermediate level of hand hygiene implementation [13].

In 2010, the University of Geneva Hospitals and Facutly of Medicine WHO Collaborating Centre on Patient Safety, in collaboration with Aesculap Academy, launched the Hand Hygiene Excellence Award (HHEA) to further support ongoing global efforts to improve hand hygiene [14]. The HHEA was designed to encourage healthcare facilities worldwide to become benchmarks for hand hygiene excellence. More than ten years after the launch of the HHEA programme, it is crucial to evaluate its implementation, and identify key drivers to further improve hand hygiene practices.

The study’ primary objective is to assess the extent of hand hygiene implementation in HHEA-award-winning healthcare facilities using the HHSAF. It intends to delineate how each element of the MMIS contributes to these practices. The study examines the relationship between HHSAF scores, hand hygiene compliance rates, and alcohol-based handrub (ABHR) usage to determine if ABHR consumption can serve as a reliable indicator of effective hand hygiene practices.

## Materials and methods

### Overview of the hand hygiene excellence award

The HHEA was conceived as a platform to identify, honour, and celebrate healthcare facilities and IPC working groups that contribute to improving safe patient care through excellence, and innovative hand hygiene multimodal promotion. Originally launched in the Asia-Pacific region in 2010, followed by Europe in 2013 and Latin America in 2014, the HHEA is currently expanding into the Middle East and Africa. Participation is open to all healthcare facilities that have successfully integrated the WHO hand hygiene MMIS, demonstrating at least 3 years of sustainability and with a documented history of sustained improvement and decreasing HAI rates. Facilities with strong leadership committed to hand hygiene as a key aspect of patient safety and quality are encouraged to apply.

The application procedure requires that hospitals complete and submit the HHSAF [7]. The HHSAF is a self-administered questionnaire designed to evaluate the hand hygiene programmes, resources and practices within healthcare facilities. The HHSAF has been validated to assess the level of hand hygiene implementation and consists of 27 indicators (each 10 to 50 points), distributed across five MMIS (see Supplementary File 1 for interpretation of MMIS elements and scoring) [8], elements (each 100 points: System Change, Training and Education, Evaluation and Feedback, Reminders in the Workplace, and Institutional Safety Climate). The maximum overall score for the HHSAF is 500 points and based on this score, a hand hygiene level is assigned to the healthcare facility, ranging from inadequate (1-125 points), basic (126–250), intermediate (251–375), or advanced (376–500; Supplementary File 1).

**Fig. 1:**
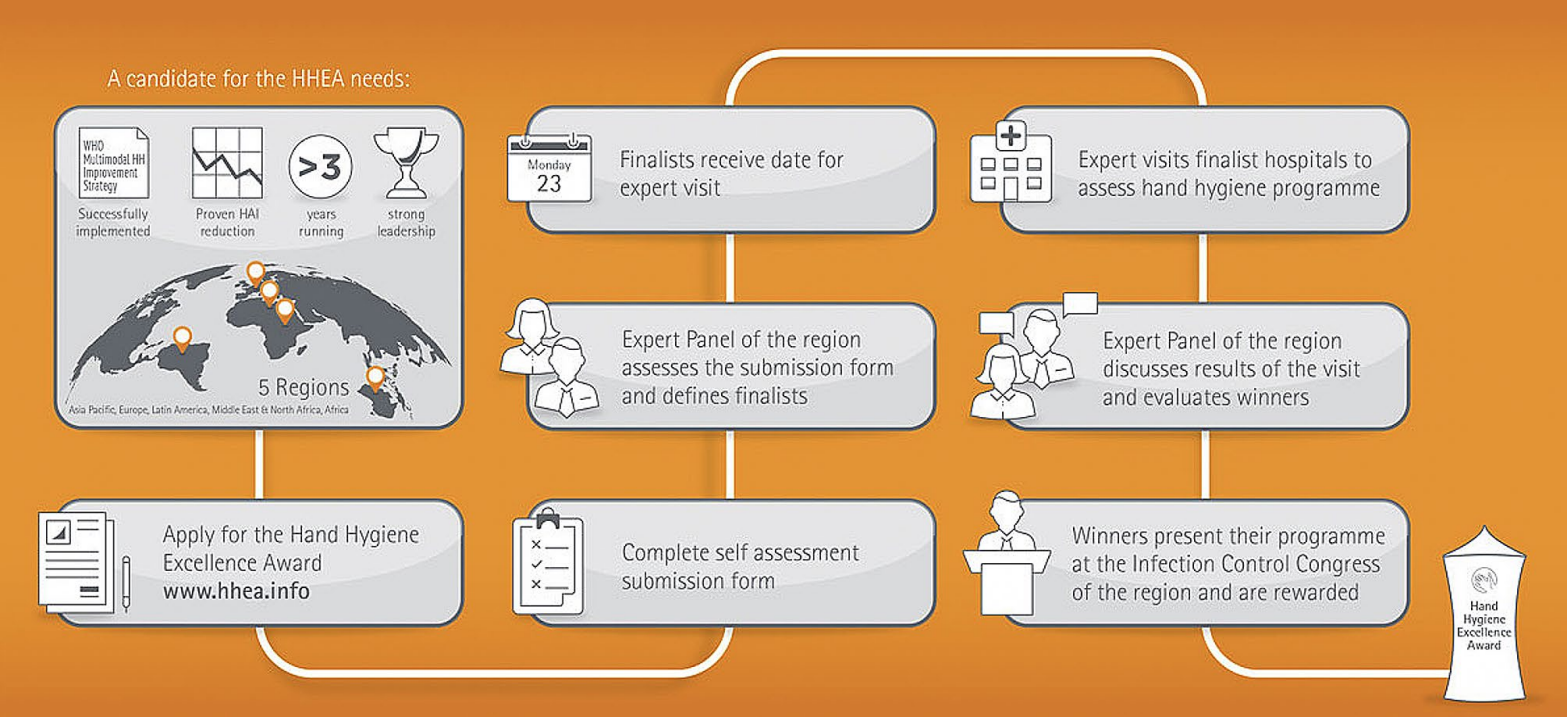
Overview of the application and selection process for the hand hygiene excellence award

A panel of international IPC experts then reviews the application forms, and consequently scores and ranks the finalists for a hospital visit. These experts are selected based on years of full-time experience and commitment to the field of IPC. Their participation is voluntary, ensuring that assessments are totally non-biased with the highest standards of professionalism. The selected healthcare facilities undergo a rigorous one-day audit conducted by two members of the HHEA expert panel (see Acknowledgments). Audit visits include interviews with hospital staff, patients and relatives, as well as a comprehensive evaluation of hand hygiene practices and infrastructures according to the HHSAF (Fig. 1). The audit process includes visits to hospital wards and departments to evaluate: (i) hand hygiene compliance, implementation of hand hygiene promotion strategies (including the use of WHO Hand Hygiene tools) and accessibility of information on hand hygiene to all staff; (ii) availability of hand hygiene agents and products - including ABHR (at the point of care and in common areas), soap, dispensers and disposable towels - and ease of accessibility of key elements (including the placement of sinks, dispensers and drying machines); (iii) presence of any form of public information or reminders of the importance of hand hygiene.

The expert panel provides advice and guidance to the finalists on ongoing efforts, sustainability, and outcome measures and finally award a winning hospital. Winners are then invited to participate in the respective regional IPC conference– that is, according to the region, the Asia-Pacific Society of Infection Control Congress (APSIC, for Asia-Pacific), the International Conference on Prevention & Infection Control (ICPIC, for the European region), or the Panamerican (or Brazilian on some occasions) Congress of Infection Control (for Latin America)– to present their hand hygiene improvement strategy and main results, and to receive the award trophy.

### Data collection and management

This study was based on datasets from HHEA forms, which included data on hand hygiene compliance, ABHR consumption, and HHSAF scores. Given the available data format, the statistical unit was represented by the single application of a hospital for the HHEA. Annual data were available for the correlation between ABHR and hand hygiene compliance. All observations were considered independent, with the assumption that this would not affect the relationship between the two variables considering the homogeneity of data collection (in conformity with international standards).

As the study did not involve any individual patient data, no informed consent was required. Hospital characteristics and any other data were retrieved in aggregated forms only, in conformity with the Helsinki Declaration and the International Regulations (EC/2016/679) concerning data protection and privacy.

### Statistical analysis

Given the skewed distribution of most indicators, descriptive statistics for quantitative variables were represented as medians and interquartile ranges (IQRs). Two-group comparisons were performed using the Mann-Whitney-Wilcoxon U test. The Kruskal-Wallis test was used for 3-group comparisons; Dunn’s post-hoc pairwise test was applied in the event of a significant result. Categorical variables are represented as absolute frequencies and percentages, and comparisons were performed using Fisher’s exact test.

Because of the structure of the HHEA form, hand hygiene compliance and ABHR consumption data were reported for the years preceding the HHSAF data. Hence, to address the lag between data measured by different indicators, estimates of ABHR and hand hygiene data corresponding to the year of the monitoring of the HHSAF were obtained through a prediction based on a linear regression trend of available data. The correlation between hand hygiene compliance and ABHR consumption, and between each of these two variables and the HHSAF score, was quantified through Kendall’s tau, and the existence of a possible non-linear relationship between ABHR consumption and hand hygiene compliance was explored through Locally Estimated Scatterplot Smoothing (LOESS) regression (Table 1).

Furthermore, considering the importance of HHSAF as a measure of hand hygiene reporting, we investigated the possible relationship between ABHR consumption and HHSAF scores using logistic regression models, where ABHR consumption was set as a dichotomous response variable (high versus low consumption), whilst the HHSAF score was treated as a continuous explanatory variable. In the absence of an evidence-based cut-off to categorise ABHR consumption, apart from the HHSAF’s implicit recommendation to use at least 20 L of ABHR per 1000 patient-days [7], we followed a methodology deployed for regional data [15] and built a sequence of logistic regression models, considering each of the possible integer values in the central tertile of ABHR observations as a cut-off to dichotomise ABHR consumption (high versus low) and in an attempt to predict this outcome from the HHSAF score.

Finally, the presence of possible ABHR cut-offs discriminating groups with significantly different HHSAF score values was also inspected using a tree-structured recursive partitioning model built using the R package “partykit”, version 1.2–20 [16]. For all analyses, *p*-values ≤ 0.05 were considered significant, even though, *p*-values between 0.05 and 0.10 were considered suggestive of a trend. All computations and plotting were performed using the statistical software R (Version 4.3.1; R Foundation for Statistical Computing, Vienna, Austria) [17].

## Results

### Descriptive framework

A total of 97 healthcare facilities applying for the HHEA from 28 countries in three world regions were included in this analysis: Latin America (60/97, 61.9%), Asia-Pacific (23/97, 23.7%), and Europe (14/97, 14.4%). Brazil and Ecuador had the greatest number of participating hospitals (24 and 18, respectively), while most countries contributed 1–2 hospitals each (a detailed prospect is available in Supplementary Table S1). The type of facility management was reported for 85 (87.6%) of them: the majority were public, university teaching hospitals (58.8%, 50/85), while private institutions accounted for the remaining 41.2% (35/85). The relative composition of the set did not vary substantially across years, except for a greater percentage of private hospitals participating in recent years (Supplementary Table S2). Participating hospitals were of various sizes (median: 235 beds), although the size varied across regions, with a tendency for smaller hospitals in Latin America and larger in the Asia-Pacific region (Table 2).

**Table 1:** Overview of the HHEA data collection process. All data reported on HHEA application forms are collected at an aggregate, healthcare facility level, hence no further detail is available for stratified analysis. More details about the HHSAF can be found in the Additional File 1

Responsible profile	Variable/task performed	Collected data	Data interpretation
IPC nurses or IPC physicians from the healthcare facilities	Data collection:		
	1) Hand hygiene compliance	Proportion (%) of opportunities in which correct hand hygiene procedures are performed	□ Range: 0–100% □ Expected target: higher compliance □ No internationally recognised threshold
	2) Alcohol-based hand rub (ABHR) consumption	Litres of ABHR used in the whole hospital, divided by the number of total bed-days (based on yearly data)	□ Expected target: higher consumption □ No internationally recognised threshold (except for a standard implicitly suggested by the HHSAF: 20 L per 1000 patient-days)
	3) Hand Hygiene Self-Assessment Form (HHSAF) score	Composite score summarising the 5 domains of the multimodal hand hygiene intervention strategy (MMIS)	□ Range: 0–500 points □ Hand hygiene level thresholds: ▢ > 375 pts: Advanced ▢ 251–375: Intermediate ▢ 126–250: Basic ▢ 0-125: Inadequate
HHEA expert panellists	One-day auditing in healthcare facilities	No data (check only performed on hospitals’ data to ensure reported standards are met)	-

**Table 2:** Descriptive characteristics of the included healthcare facilities participating in the HHEA. HHSAF data refers to years 2017–2023, while ABHR consumption data and average compliance include figures from 2012 to 2021. Healthcare facilities are taken as statistical units, and data are presented as medians and IQRs for the overall set, as well as stratified by management type and by region. Multiple comparisons between regions were performed in the event of a *p* < 0.10 after the Kruskal-Wallis test

Variable	Overall	Management						
		General/University/Teaching	Private	*p*-value	Asia-Pacific	Europe	Latin America	*p*-value
Size (number of beds)	235 [128–441]	320 [206–672]	148 [90–253]	< 0.001	550 [252–1260]	362 [164–509]	161 [107–320]	< 0.001^†^
ABHR consumption (average, in ml/PD)	31.9 [19.7–62.7]	31.9 [18.4–63.6]	31.3 [21.3–63.2]	0.901	24.1 [10.4–39.0]	34.0 [21.5–55.7]	42.3 [22.0–66.7]	0.087^#^
Average hand hygiene compliance (%)	71.6 [59.2–80.6]	67.4 [53.1–80.3]	70.7 [66.3–78.9]	0.502	72.4 [69.7–80.0]	79.3 [72.0–82.4]	65.8 [55.1–80.2]	0.057^§^
WHO HHSAF								
System change	100 [95–100]	100 [80–100]	100 [100–100]	N.A.*	100 [100–100]	100 [100–100]	100 [85–100]	N.A.*
Education & Training	95 [80–100]	95 [82.5–100]	90 [80–100]	0.336	100 [92.5–100]	95 [80–100]	90 [80–100]	0.086^#^
Evaluation & Feedback	90 [80–95]	85 [72.5–95]	95 [85–95]	0.056	95 [87.5–100]	87.5 [80–95]	85 [75–95]	0.023^#^
Reminders in workplace	90 [80–100]	90 [80–95]	90 [67.5–100]	0.388	95 [87.5–100]	95 [82.5–100]	87.5 [67.5–95]	0.026^#^
Safety Climate	85 [70–95]	85 [70–90]	90 [77.5–95]	0.436	90 [82.5–100]	80 [62.5–90]	85 [70–90]	0.031^‡^
**Total HHSAF score**	**445 [395–480]**	**435 [387.5–472.5]**	**455 [395–485]**	0.233	**475 [447.5–490]**	**442.5 [417.5–475]**	**432.5 [380–467.5]**	< 0.001^#^

^†^Significant differences were found between Latin America and Europe, and between Latin America and Asia-Pacific

^#^A significant difference was found between Latin America and Asia-Pacific

^§^A significant difference was found between Latin America and Europe

*The Kruskal-Wallis test could not be performed, owing to the high number of ties (most values were equal to the maximum score of 100 points)

^‡^Significant differences were found between Asia-Pacific and Europe, and between Asia-Pacific and Latin America

**Fig. 2:**
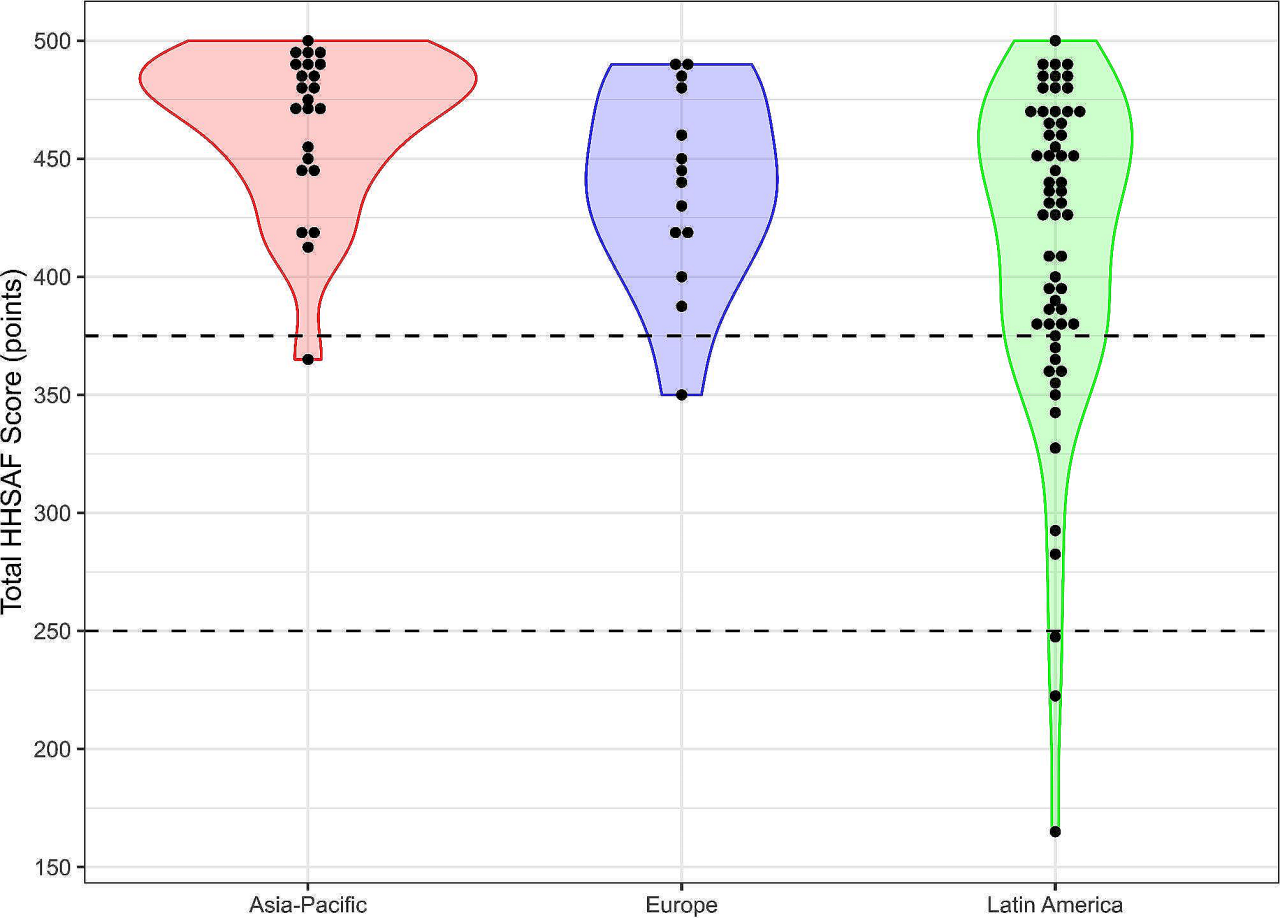
Violin plots of the distribution of HHSAF total scores by region. Each dot (statistical unit) represents a HHEA winning healthcare facility. Dashed lines indicate the basic/intermediate (250 points) and intermediate/advanced (375 points) thresholds established by the HHSAF 2010

Overall hand hygiene implementation, as indicated by the HHSAF scores, was strong, with a high median total HHSAF score (445 points, IQR 395–480) while 81 healthcare facilities (83.5%), reached an advanced hand hygiene implementation level (> 375 out of 500 points (Supplementary File 1, HHSAF score and hand hygiene level). The HHSAF scores reached the highest levels in 2019 and 2021 (median scores: 480 and 470 points, respectively); however, no data for this parameter were available for 2020. The distribution of total HHSAF scores is depicted in Fig. 2: regional differences in the totals were observed (Table 2), with the Asia-Pacific scoring highest (median 475, IQR 447.5–490), followed by Europe (median 442.5, IQR 417.5–475) and Latin America (median 432.5, IQR 380-467.5).

Considering each of the MMIS elements separately, successful implementation of a system change was observed across all regions and participating facilities, as evidenced by the fact that 68 facilities (70.1%) achieved the highest possible score for this element. Conversely, regional differences appeared in the scores obtained for other MMIS elements: the Asia-Pacific region steadily stood out for the highest scores in each element, particularly regarding education and training, evaluation and feedback, and safety climate.

Details of each MMIS element as well as stratification by geographic area and management type are presented in Table 2 (results by region are also depicted in Supplementary Fig. S1). Notably, no significant differences were detected between the public/university and private hospitals for any of the indicators. Median hand hygiene compliance was 71.6% (IQR: 59.2-80.6%), with regional medians ranging from 65.8% in Latin America to 79.3% in Europe. ABHR consumption showed a median value of 31.9 ml/PD; unlike for other hand hygiene indicators, Latin America showed the highest use of ABHR (42.3 ml/PD), while Asia-Pacific and Europe reached a median of 24.1 and 34.0 ml/PD, respectively (Table 2). An improvement was observed on average between the pre-pandemic period (median ABHR consumption between 17.2 and 42.0 ml/PD for years 2012–2018) and the years of COVID-19 (50.9–60.3 ml/PD for years 2019–2021, with a peak corresponding to the 2020 pandemic wave).

**Fig. 3:**
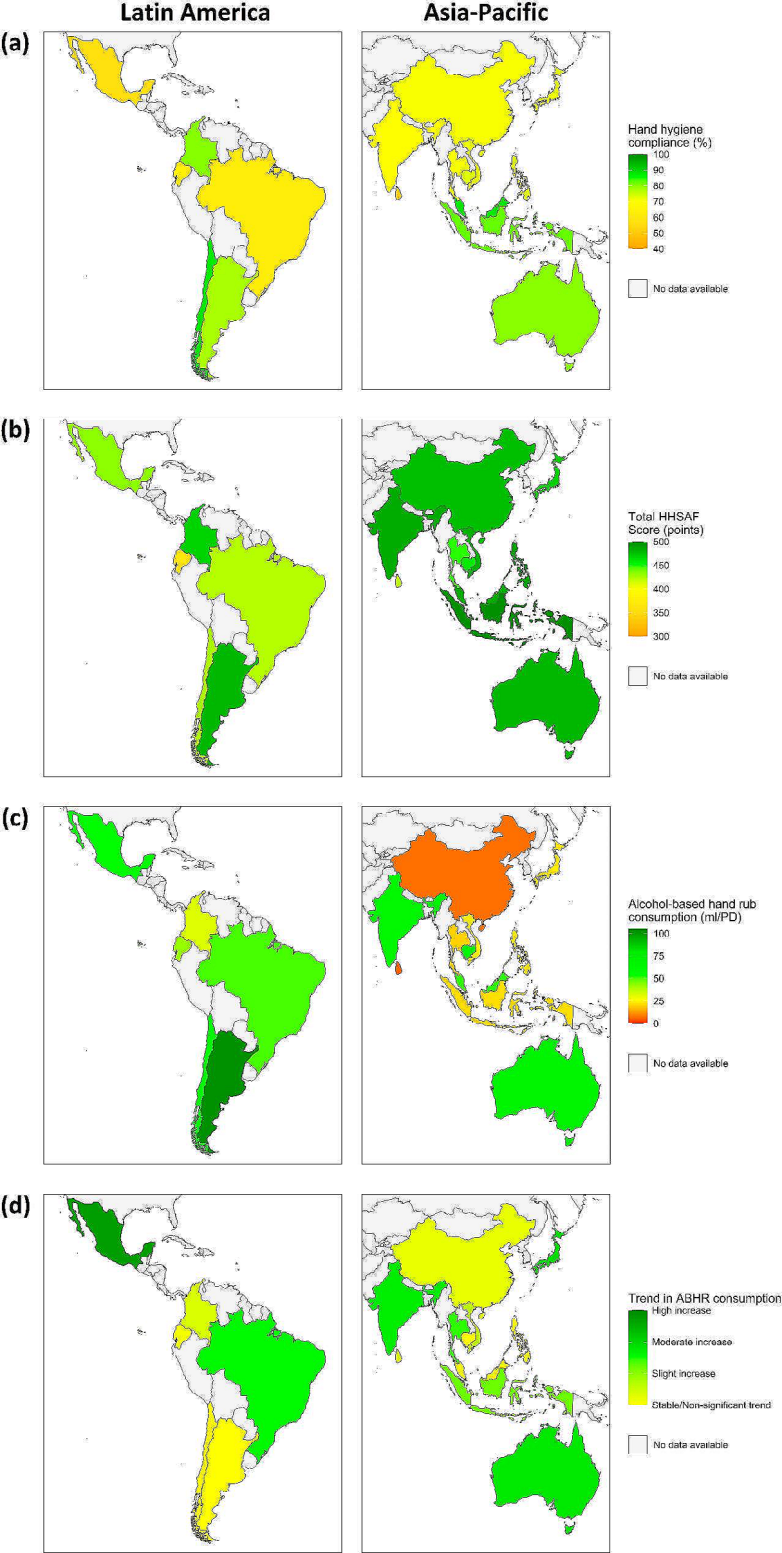
Geographical representation of average hand hygiene performances in countries participating in the global Hand Hygiene Excellence award in Asia-Pacific and Latin America. Each country is coloured according to the arithmetic mean of the hand hygiene compliance (**a**), total HHSAF scores (**b**), ABHR consumption (**c**) and ABHR consumption trend coefficients (**d**), of the respective hospitals participating in the HHEAs. This map considers 2012–2021 data for hand hygiene compliance and ABHR consumption, and 2017–2023 data for HHSAF scores. Mollweide’s projection was used to build the maps.

A map of hand hygiene performance by country in Latin America and Asia-Pacific for each parameter (hand hygiene compliance, HHSAF scores, ABHR consumption, and trends) is shown in Fig. 3. Inter-regional differences in hand hygiene compliance (Fig. 3a) emerged in both contexts, with lower average levels (< 70%) in Brazil, Mexico, Ecuador (Latin America), Sri Lanka and India (Asia-Pacific). HHSAF scores were high across all countries (> 400 points, Fig. 3b), while a lower average ABHR consumption (< 20 ml/PD) was recorded in some Asian countries (Sri Lanka, China, and Thailand, Fig. 3c). ABHR consumption trends were generally found to be increasing, with only some stable-trend exceptions in Latin America (Argentina, Chile) and Asia (Brunei, Cambodia, and Philippines, Fig. 3d).

European data were available for a limited set of countries (Supplementary Fig. S2) and showed generally high HHSAF scores, although with poorer hand hygiene compliance in Southern European countries. On average, ABHR consumption was lower in Spain and Belgium (∼18 ml/PD), and the ABHR consumption trends were more promising in Eastern Europe than in other countries.

### Correlation analyses

Data collected during the study period showed that across the participating healthcare facilities, HHSAF scores were positively correlated with hand hygiene compliance (*τ* = 0.211, *p* = 0.007), while no significant linear correlation was detected between HHSAF scores and ABHR consumption (*τ* = 0.107, *p* = 0.204). Considering all observations throughout the years, hand hygiene compliance rates were positively correlated with ABHR consumption (*τ* = 0.193, *p* < 0.001), and such correlation was significant in each of the three regions (Asia-Pacific: *τ* = 0.181, *p* = 0.009; Europe: *τ* = 0.375, *p* = 0.001; Latin America: *τ* = 0.237, *p* < 0.001).

**Fig. 4:**
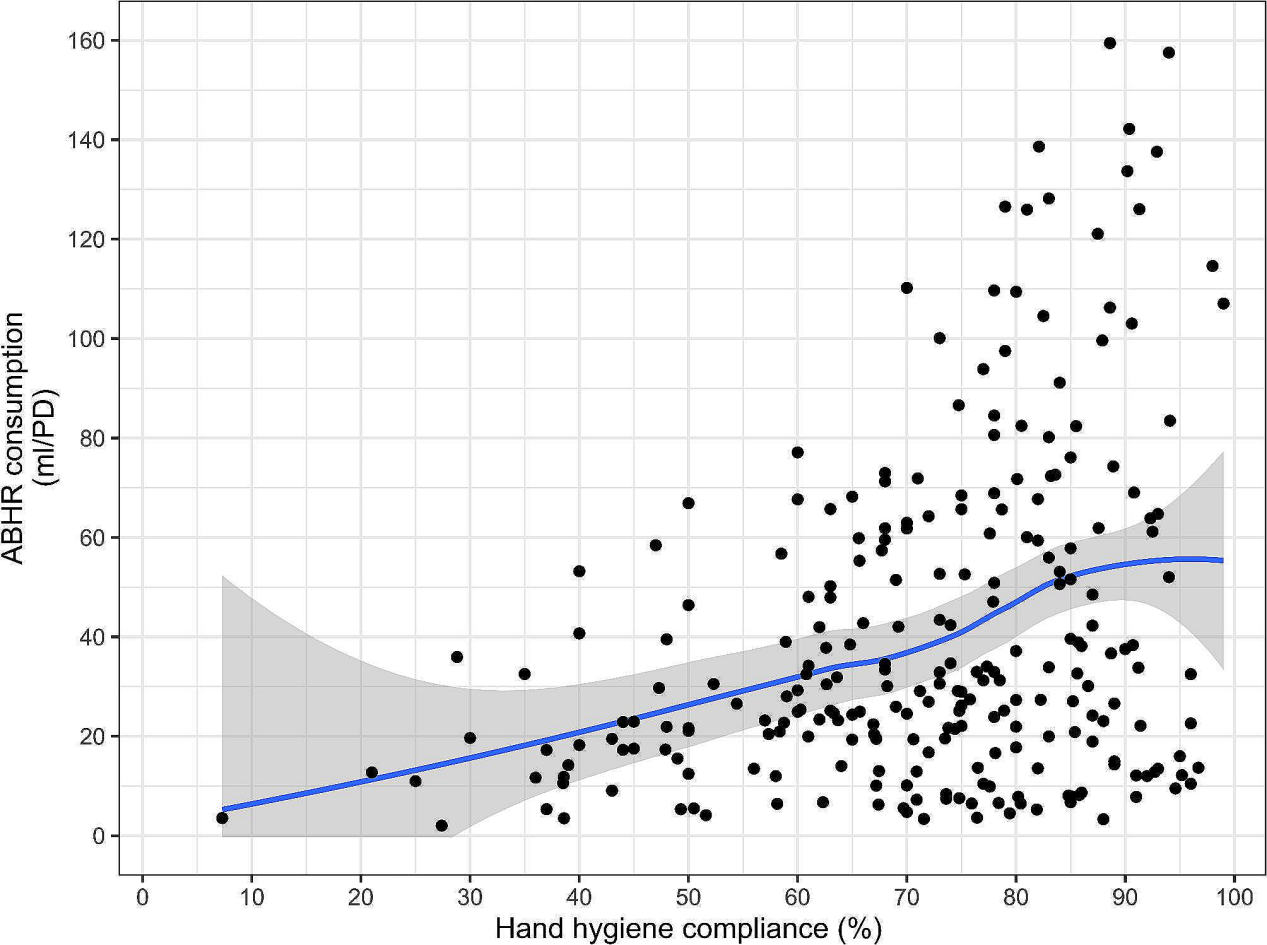
Scatterplot representation of ABHR consumption in relation to hand hygiene compliance. Each dot represents a single observation (point data from one year in a single hospital). A non-linear fit of point data (blue line) is provided, along with its 95% confidence interval (shaded area), according to the LOESS regression.

The scatterplot analysis (Fig. 4) confirmed that a higher hand hygiene compliance was generally associated with increased ABHR consumption, with a trend toward a linear relationship between the two variables, at least for compliance values up to 80%. Low hand hygiene compliance rates (below 40%) were associated with ABHR consumption values lower than 30 ml/PD, while ABHR use in the range between 30 and 60 ml/PD were associated with compliance rates of approximately 70–80%. Notably, compliance levels above 80–85% were achieved in the presence of more dispersed values of ABHR consumption, yet without a conspicuous increase in the average estimate predicted by the LOESS model (stable around 50–60 ml/PD, Fig. 4).

### Logistic regression models and partitioning trees

Considering the distribution of ABHR consumption across the healthcare facilities participating in the study, logistical models were built, as described in the Methods section (§Statistical Analysis), by setting the cut-off (to discriminate high- versus low-consumption facilities) at all possible integer values *i* in the central tertile of the observed ABHR distribution (i.e. ranging from 30 to 67 ml/PD). A full report of the models is detailed in Table 3: for any of the ABHR threshold values investigated, healthcare facilities with higher HHSAF scores had a greater probability of belonging to the high-consumption group than to the low-consumption group (with an OR varying between 1.059 and 1.124 per 10-point increase in the HHSAF score, Table 3). Importantly, this result approached statistical significance only when the cut-off was set at 58–59 ml/PD: in this case, the model predicted an increase of 12.4% [95% CI: -1.9%; +28.9%) in the odds of being in the high consumption group for every 10-point increase in the HHSAF score (Table 3).

**Table 3:** Output of the logistic regression models developed by choosing different cut-offs to discriminate high- versus low-ABHR consumption groups within the central tertile of the distribution of predicted ABHR data (cut-offs outside this range are reported in Supplementary Table S2). For each cutoff *i* of ABHR consumption (dependent variable), hospitals were divided in 2 groups (high vs. low) and the logistic regression was built as described in the Methods. The table shows the odds ratio (OR_10_) of belonging to the high-consumption group for every 10-point increase in the HHSAF score (independent variable). Values reported in bold indicate cut-off values for which statistical significance is approached (*i* = 58–59)

Cut-off (ml/PD)	OR_10_ [CI 95%]	p-value	Cut-off (ml/PD)	OR_10_ [CI 95%]	p-value
30	1.090 [0.956–1.244]	0.198	49	1.074 [0.944–1.222]	0.280
31	1.097 [0.963–1.249]	0.165	50	1.074 [0.944–1.222]	0.280
32	1.097 [0.963–1.249]	0.165	51	1.074 [0.944–1.222]	0.280
33	1.084 [0.953–1.232]	0.220	52	1.074 [0.944–1.222]	0.280
34	1.082 [0.952–1.230]	0.226	53	1.074 [0.944–1.222]	0.280
35	1.082 [0.952–1.230]	0.226	54	1.074 [0.944–1.222]	0.280
36	1.083 [0.953–1.230]	0.224	55	1.074 [0.944–1.222]	0.280
37	1.083 [0.953–1.230]	0.224	56	1.074 [0.944–1.222]	0.280
38	1.068 [0.940–1.212]	0.313	57	1.074 [0.944–1.222]	0.280
39	1.068 [0.940–1.212]	0.313	**58**	**1.124 [0.981–1.288]**	**0.092**
40	1.068 [0.940–1.212]	0.313	**59**	**1.124 [0.981–1.288]**	**0.092**
41	1.068 [0.940–1.212]	0.313	60	1.092 [0.955–1.249]	0.198
42	1.068 [0.940–1.212]	0.313	61	1.028 [0.902–1.172]	0.675
43	1.068 [0.940–1.212]	0.313	62	1.059 [0.925–1.213]	0.408
44	1.102 [0.967–1.256]	0.143	63	1.059 [0.925–1.213]	0.408
45	1.093 [0.959–1.245]	0.182	64	1.059 [0.925–1.213]	0.408
46	1.093 [0.959–1.245]	0.182	65	1.059 [0.925–1.213]	0.408
47	1.093 [0.959–1.245]	0.182	66	1.060 [0.924–1.216]	0.408
48	1.074 [0.944–1.222]	0.280	67	1.060 [0.924–1.216]	0.408

**Fig. 5:**
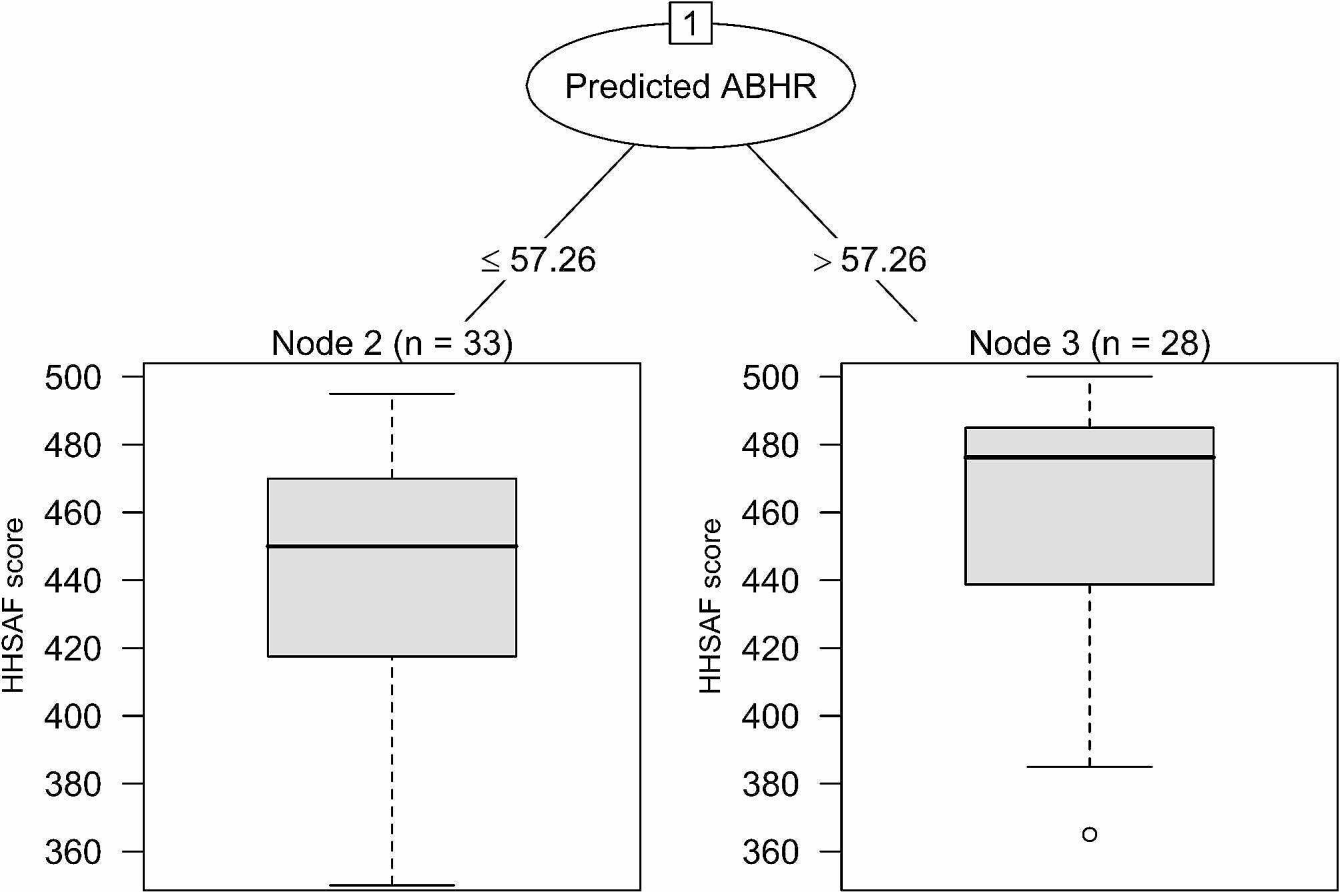
Partitioning algorithm computed by the R package “partykit” (version 1.2–20) according to HHSAF score and ABHR consumption. To ensure the presence of an appropriate sample size, a minimum number of 20 observations was required in each group (the same model without size constraints is shown in Supplementary Fig. S3). The best partition was obtained for an ABHR consumption threshold slightly above 57 ml/PD.

A similar cut-off was also chosen by the partitioning tree, which indicated that the optimal cut-off for the partition could be achieved at 57.6 ml/PD of consumed ABHR, thereby selecting two groups with median HHSAF scores of 450 [417.5–470] and 476 [444–485] points, respectively (Fig. 5). Repeating both analyses (correlation and partitioning tree) over all possible values of the ABHR distribution still led to higher ABHR thresholds (around 77–80 ml/PD), even though such a categorisation would leave few hospitals in the high-consumption group, and hence might provide less robust results owing to the limited sample of this group (see Table S3 and Fig. S3 in the Supplementary file for these additional results).

## Discussion

The Hand Hygiene Excellence Award has been a global initiative advancing hand hygiene improvement over the past decade. Ten years after its launch, this study provides an overview of the HHEA process and the level of hand hygiene implementation, including related indicators and outcomes, in 97 healthcare facilities across 28 countries worldwide. Unsurprisingly, the majority of healthcare facilities demonstrated a high level of hand hygiene implementation (median 445 points, IQR: 395–480), well above the advanced threshold (375 points) established by the WHO HHSAF, which is consistent with a sample of centres applying for a hand hygiene excellence award, thus potentially playing a leading role as testimonials for promotion campaigns. As anticipated, the hand hygiene indicators recorded a substantial increase during the years when the COVID-19 pandemic was prevalent (2019–2021), with HHSAF scores approaching the highest possible level, and the consumption of ABHR showing a two- or threefold increase compared to baseline data.

The highest-scoring element of the MMIS through the HHSAF was system change, with decreasing scores for the following elements until reaching the lowest score for institutional safety climate. This highlights the greater challenge in creating a safety climate, compared to availability of infrastructure and supplies, and emphasizes the need for further focus on this crucial aspect to promote clean and safe care [13]. This finding aligns with previous research, which identifies a systemic issue in cultivating an organizational culture that truly values and pursues organization-wide enhancements in hand hygiene practices [13, 18–20]. Potential hindrances to hand hygiene improvement include lack of patient engagement, disconnected leadership, and absence of perceived [20, 21]. To address these challenges, healthcare facility leadership must foster a culture of safety that prioritizes hand hygiene improvement at all levels and consistently models and reinforces hand hygiene behaviour [21, 22]. While patient involvement in hand hygiene promotion poses challenges, it can provide an additional layer of accountability and encouragement for health workers [23].

The analysis by region showed greater achievements in the Asia-Pacific region. This finding may be attributed to factors, including cultural and traditional elements, organisational context, regional policies, or promotion of hand hygiene initiatives, even considering the occurrence of viral respiratory outbreaks [24, 25]. The difference in the size of healthcare facilities may also play a role, as hospitals in the Asia-Pacific had a greater number of beds (Table 2) and, potentially a higher degree of care complexity [26–29]. The impact of the HHEA initiative, first launched in the Asia-Pacific region (2010) than in Europe (2013) and Latin America (2014) may also contribute to these findings [30]. This promising result encourages the expansion of international campaigns to unreached regions.

The median hand hygiene compliance level reported in our study was 71.6%, which appears to be relatively high when compared to broader literature. However, it’s important to note that our study sample comprised healthcare facilities recognized for their excellence in hand hygiene practices, as evidenced by their receipt of the hand hygiene excellence award. This specific cohort of healthcare facilities is likely to exhibit superior performance in hand hygiene compliance, which may explain the high compliance rates. The outcomes of our study align with the expected oucomes for centers applying for a hand hygiene excellence award and suggest that these facilities could serve as leaders in hand hygiene promotion. Although the high levels of compliance we report may surpass the typical findings, especially in low-income countries where compliance is lower, they are aligned with the anticipated outcomes for healthcare facilities distinguished by their excellence in hand hygiene practices [3].

Increased ABHR consumption is associated with higher levels of hand hygiene compliance across healthcare facilities and this relationship is consistent in all three regions, confirming that ABHR availability plays a fundamental role in hand hygiene promotion. The WHO HHSAF recommends a consumption target of at least 20 L of ABHR per 1000 patient-days (i.e. 20 ml/PD) [31]. This recommendation was based on the average consumption of ABHR monitored in 1998 at the University of Geneva Hospitals (HUG) after 3 years of implementation of the multimodal hand hygiene promotion strategy that led to the development of the WHO MMIS [9]. Today, annual ABHR consumption levels at HUG average 60–80 ml/PD, with high-intensity care wards [32] such as intensive care units using more. Given that the benchmark of 20 ml/PD for ABHR consumption was established over 25 years ago, based on average values monitored in a large referral healthcare facility various types of wards, it is important to recognize that this target may not be suitable for individual wards or healthcare facilities with differing levels of patient care activity, requiring varying frequencies of hand hygiene opportunities corresponding to the intensity of patient care [32]. The higher the intensity of patient care activities, the higher the number of opportunities for hand hygiene [9, 32, 33]. Consequently, it is possible that the current target may be insufficient to achieve optimal hand hygiene compliance levels, which are expected to be higher than 40%. Previous studies have emphasized the need for re-evaluation of the 20 ml/PD ABHR threshold [15, 34]. Borg et al. reported a reduction in healthcare-associated MRSA bacteraemia when ABHR consumption reached 40 mL/PD [35]. It is also worth noting that ABHR consumption levels in high-intensity-of-care wards, such as intensive care units, can reach 100 to 160 ml/PD in facilities with proven high levels of hand hygiene compliance (DP, personal communication).

The data suggest that predicting ABHR consumption based on compliance is less reliable at high hand hygiene compliance levels (Fig. 2) hinting at the influence of other factors. A plateau in ABHR consumption beyond 80–85% hand hygiene compliance levels, points to the importance of additional factors over ABHR use alone. Key contributors to achieving an 80% (or higher) hand hygiene compliance, include leadership, standardised national approach with authority engagement, and transparent reporting of hand hygiene compliance data [10]. As hand hygiene compliance improves, incorporating organisational and behavioural strategies becomes crucial due to the complex nature of hand hygiene improvement and sustainability [36, 37].

The plateau observed in the presence of an average predicted value of approximately 55 ml/PD might suggest an optimal level of ABHR use. This finding is supported by the partitioning algorithm and the logistic regression models, which both set optimal thresholds between 55 and 60 ml/PD. This cut-off is higher than the one (23 ml/PD) detected in a similar study [15], likely due to the markedly skewed distribution of ABHR consumption, with most hospitals not exceeding 20 ml/PD. Moreover, values around 55–60 ml/PD were closer to the actual ABHR consumption observed in contexts where hand hygiene was considered an absolute priority such as during the COVID pandemic [25] or nosocomial outbreaks [38]- and may thus represent a reasonable cut-off for clinical practice.

ABHR consumption is an important indicator of hand hygiene compliance and can serve as a valid proxy measure, at least for compliance values up to 80–85% [39]. The HHSAF is confirmed as a reliable measure for hand hygiene, correlating with hand hygiene compliance rates and the level of IPC implementation. Higher total HHSAF scores have shown higher hand hygiene compliance rates [15, 40]. A global survey showed a positive correlation between the HHSAF and the level of implementation of the IPC Core Components as measured by the WHO IPC Assessment Framework (IPCAF) scores [13] in 4440 acute healthcare facilities across 81 countries worldwide [41]. Higher HHSAF scores were associated with higher IPCAF scores, which reinforces the validity of the HHSAF tool and highlights the importance of hand hygiene as a predictor of overall IPC level in healthcare facilities. Additionally, the HHSAF has shown potential in discerning high versus low ABHR consuming facilities and can predict with a degree of probability which group a facility might belong to. These findings support the continued use of HHSAF as a process measure to monitor and enhance hand hygiene practices progressively [15, 41, 42].

This study has limitations. There is a potential for bias in the HHSAF due to the convenience sampling [43], as facilities participating in the HHEA may be already invested to hand hygiene initiatives, which may lead to overestimation in the data. Nevertheless, the breadth of data across key indicators permitted a robust statistical analysis. The self-reporting aspect of the HHSAF, and potential shortcomings in the hand hygiene compliance data, may impact the strength of inferred associations. However, the credibility of HHSAF scores is enhanced by the HHEA programme’s audit component. The absence of certain aggregated data, such as ICU bed numbers and limited information of long-term care wards, restricted a more stratified analysis for the degree of healthcare complexity. Despite these constraints, the study’s findings remain relevant across three global regions, suggesting that the models developed have broad applicability. Robust statistical methods were used to examine the linear and non-linear associations between hand hygiene indicators (i.e. HHSAF scores, hand hygiene compliance, and ABHR consumption), reinforcing the study’s conclusions.

## Conclusion

This study demonstrates progress in hand hygiene implementation through HHEA across 97 healthcare facilities in 28 countries. The majority of these facilities have achieved hand hygiene levels above the advanced threshold established by the HHSAF. The study’s findings are based on a validated HHSAF tool that enables the benchmarking of hand hygiene implementation over time and place. While system change ranks highest in MMIS, there is an ongoing challenge in fostering a supportive institutional safety climate, which requires focused leadership and patient engagement. This study has highlighted notable successes in three regions worldwide and it suggests that ABHR consumption should be considered along with other hand hygiene determinants. The findings contribute to the discourse on improving hand hygiene in healthcare settings and offer insights that inform future patient safety policy updates.

### Electronic supplementary material

Below is the link to the electronic supplementary material.

Supplementary Material 1

## Data Availability

Data is provided within the manuscript and the supplementary files.

## Abbreviations

AMR: Antimicrobial resistance
HAIs: Healthcare-associated infection
HHEA: Hand hygiene excellence award
IPC: Infection prevention and control
MMIS: Multimodal improvement strategy
WHO: World Health Organization
