# Supplementary material for: Ten years of hand hygiene excellence: a summary of outcomes, and a comparison of indicators, from award-winning hospitals worldwide
Source: Antimicrob Resist Infect Control. 2024 Apr 19;13:45. doi: 10.1186/s13756-024-01399-0 (PMC11027265; doi:10.1186/s13756-024-01399-0)

**Supplementary Information**

Supplement to: **Ten Years of Hand Hygiene Excellence Award: Indicators and outcomes from award-winning hospitals**

**CONTENT**

1. **Additional file 1: WHO Hand Hygiene Self-Assessment Framework**
2. **Additional file 2: Supplementary Tables**

**Table S1.** Countries and regions of participating healthcare facilities.

**Table S2.** General characterisation of the hospital participating in the HHEAs.

**Table S3.** Output of the logistic regression models developed by choosing different cut-offs to discriminate high- versus low-ABHR consumption groups outside the central tertile of ABHR distribution (values in the central tertile are reported in Table 3 of the main manuscript).

1. **Additional file 3: Supplementary Figures**

**Figure S1.** Distribution of scores for single MMIS elements of the HHSAF by region.

**Figure S2.** Geographical representation of average HH performances in countries participating in the global Hand Hygiene Excellence Awards in the European region.

**Figure S3.** Partitioning algorithm computed by the R package “partykit” (version 1.2-20) according to HHSAF score and ABHR consumption, with no group size constraint.

**Additional File 1: WHO Hand Hygiene Self-Assessment Framework**


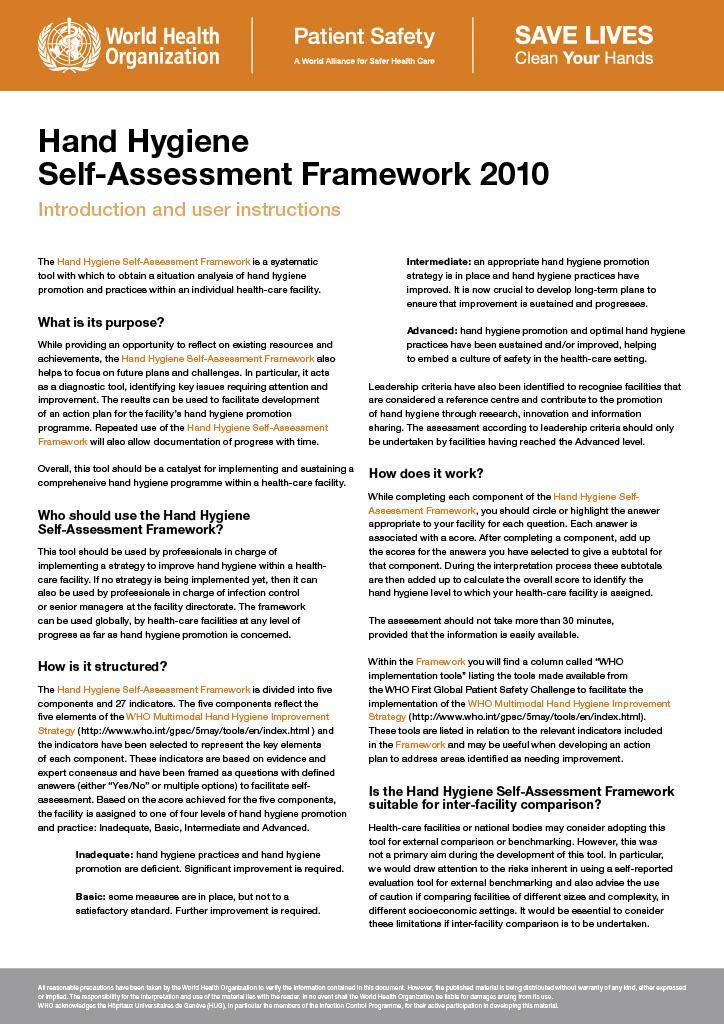


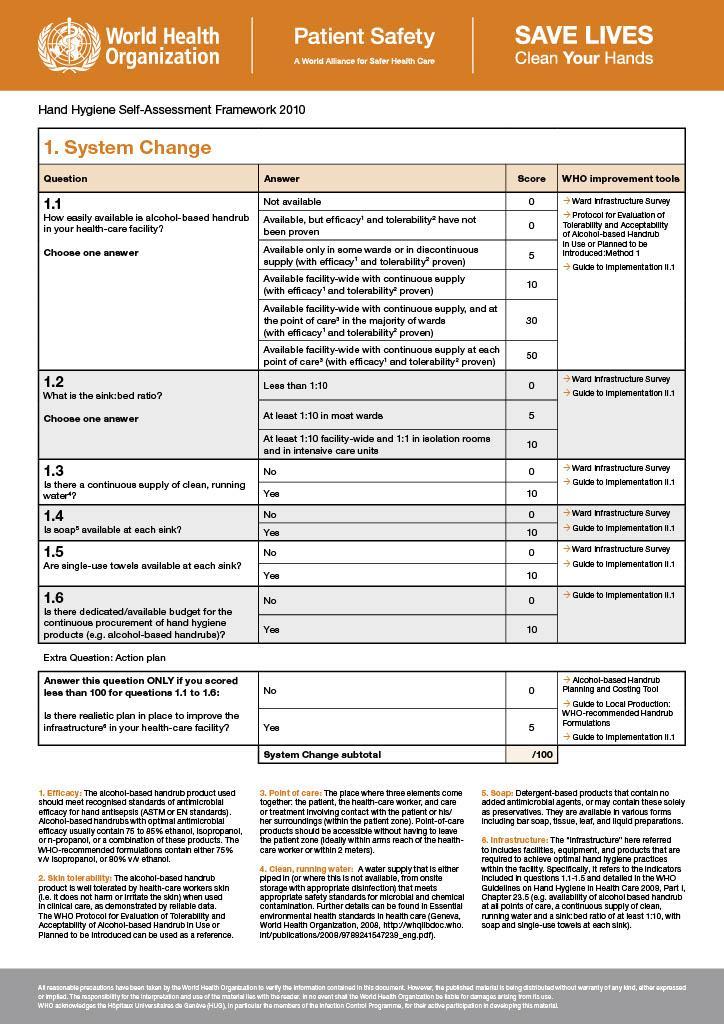

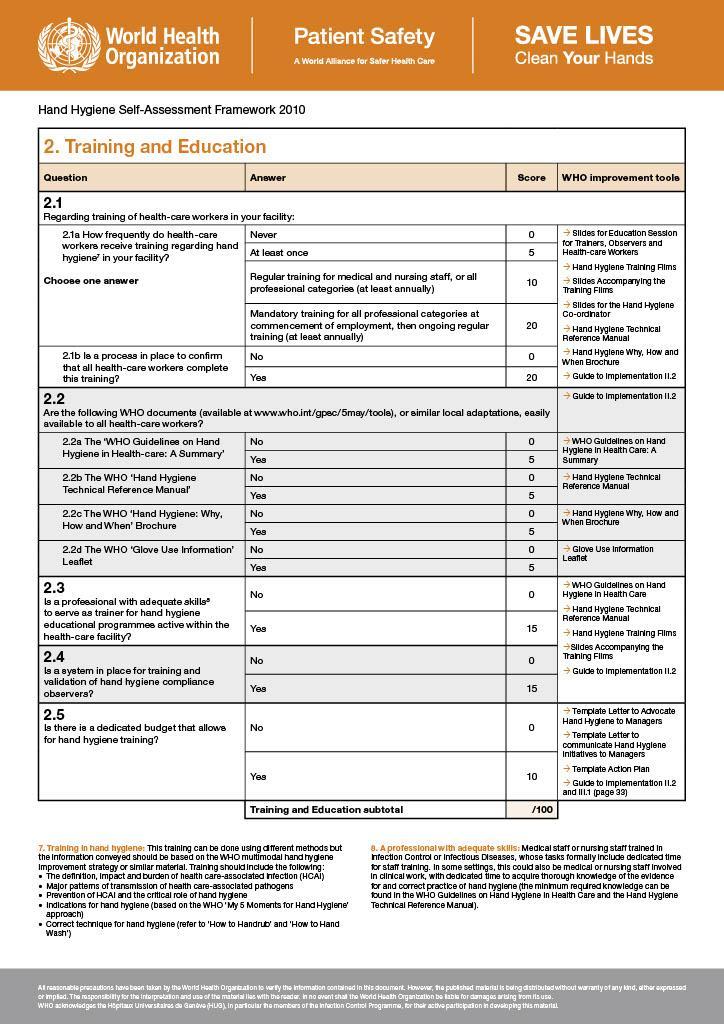

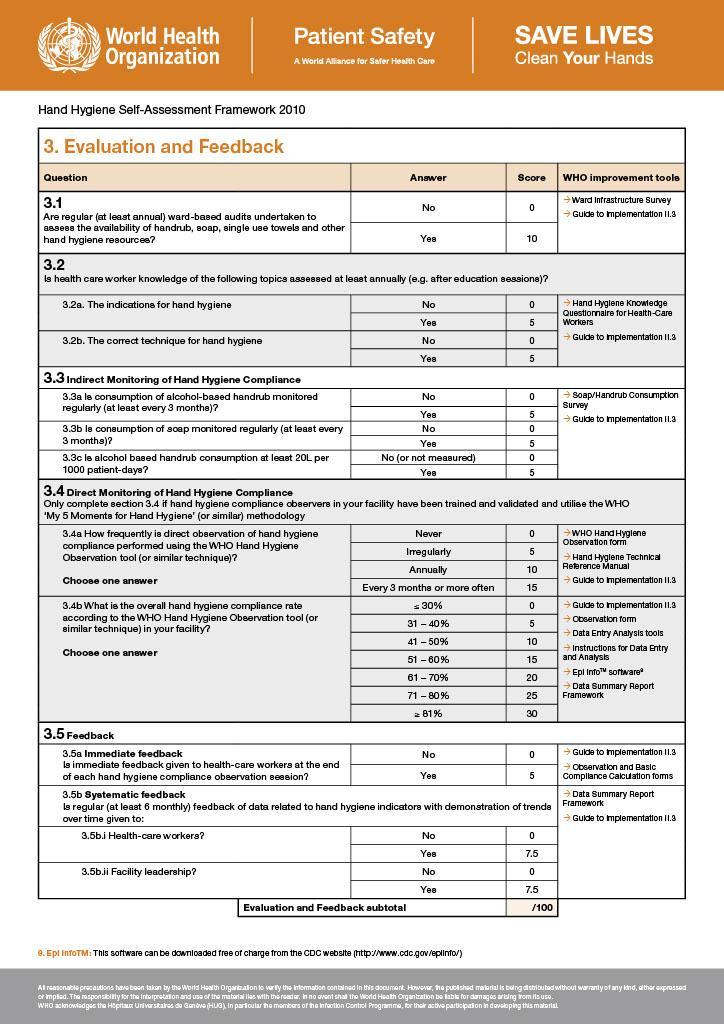

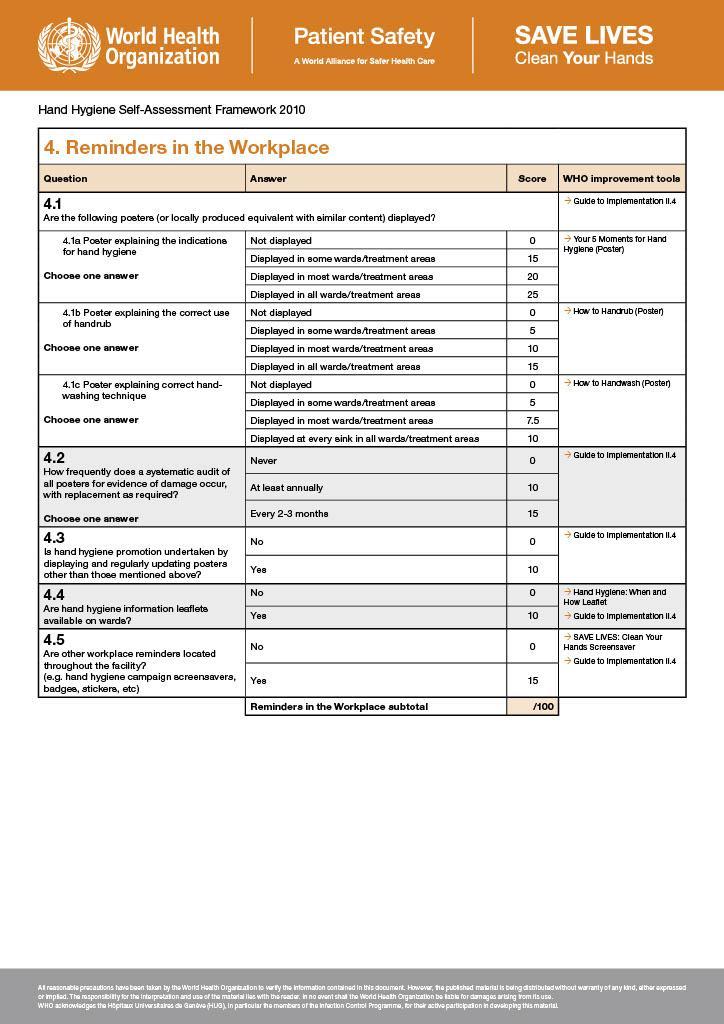

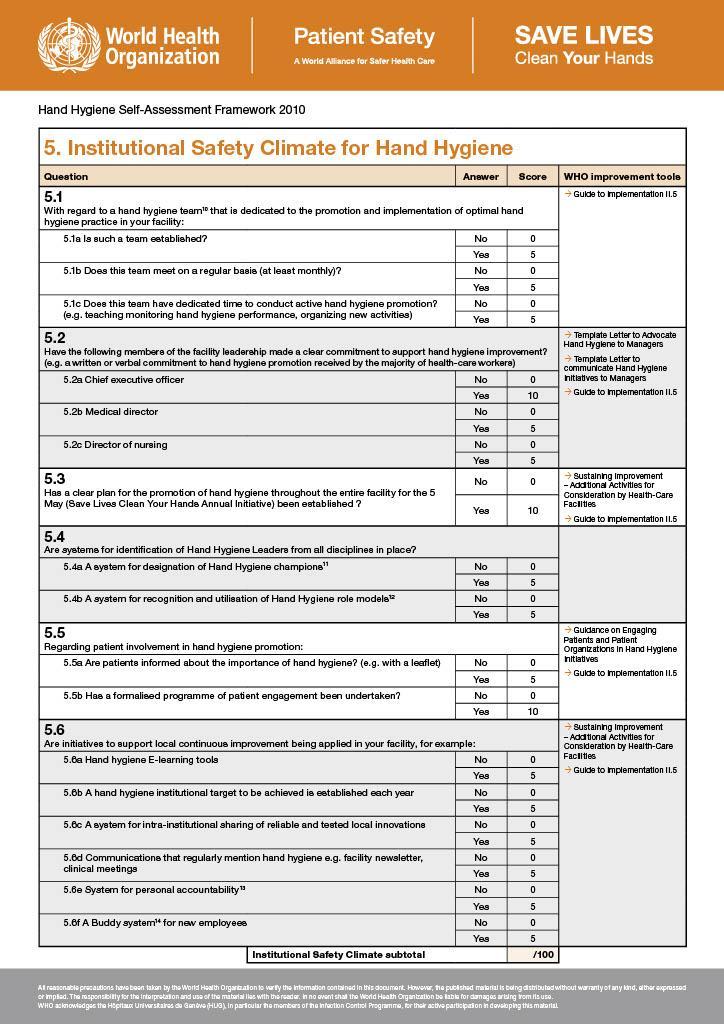

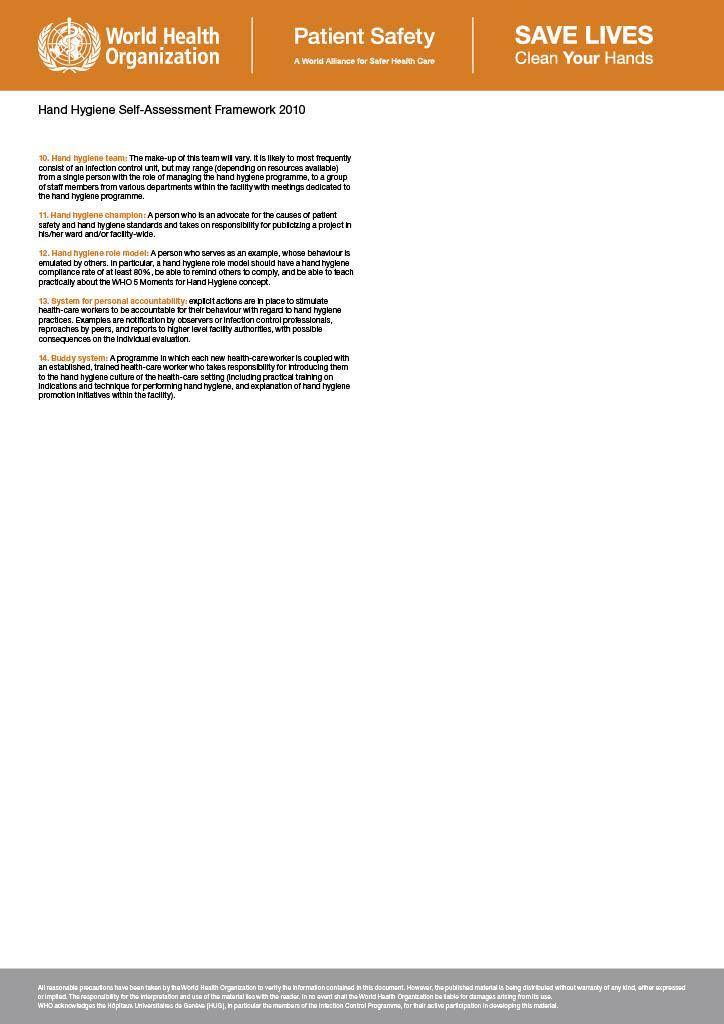

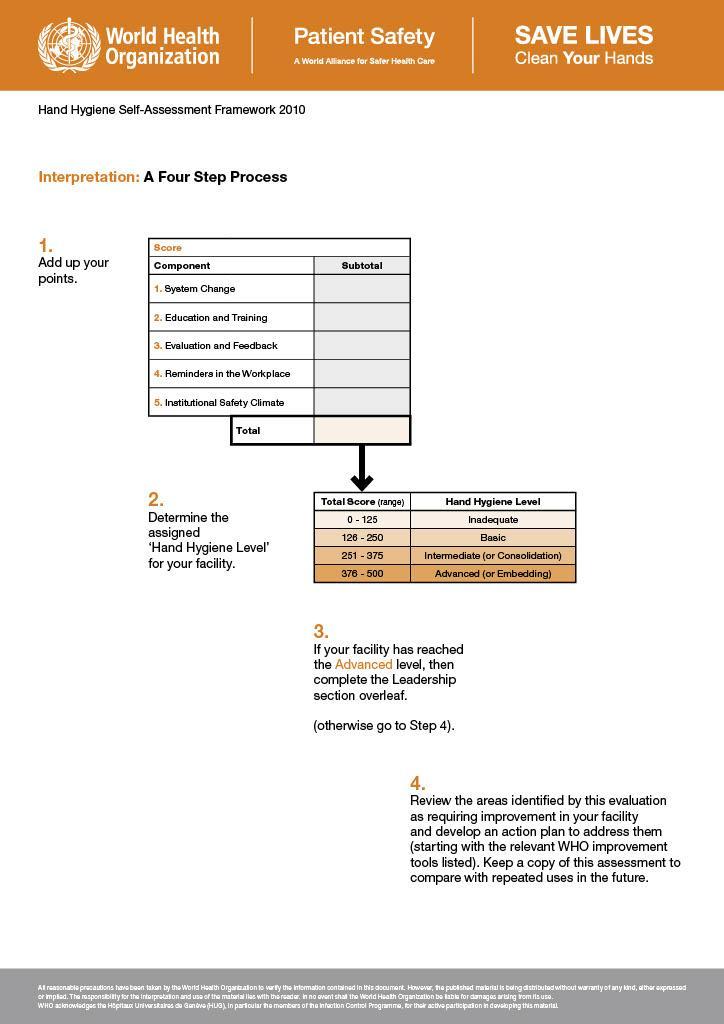

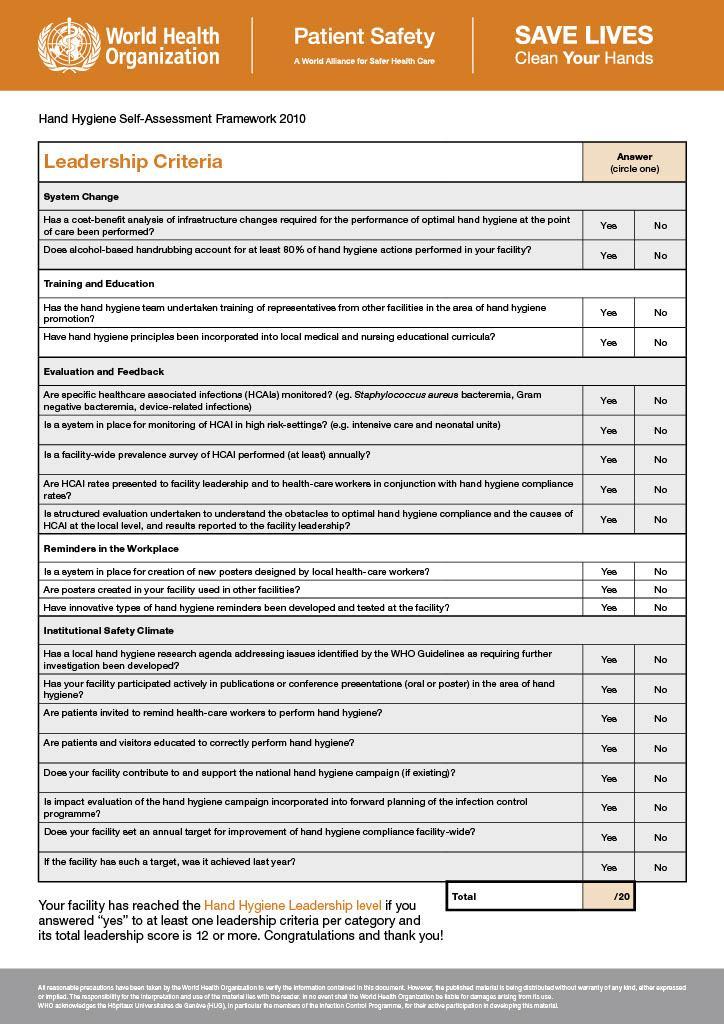


**Additional File 2: Supplementary Tables**

**Table S1. Countries and regions of participating healthcare facilities**

| **Asia-Pacific** | | **Europe** | | **Latin America** | |
| --- | --- | --- | --- | --- | --- |
| **Country** | **Number of participating hospitals** | **Country** | **Number of participating hospitals** | **Country** | **Number of participating hospitals** |
| Thailand | 5 | Czech Republic | 3 | Brazil | 24 |
| Malaysia | 3 | Switzerland | 3 | Ecuador | 18 |
| Cambodia | 2 | Belgium | 1 | Mexico | 10 |
| China | 2 | Denmark | 1 | Colombia | 5 |
| Indonesia | 2 | Ireland | 1 | Chile | 2 |
| Turkey | 2 | Portugal | 1 | Argentina | 1 |
| Viet Nam | 2 | Romania | 1 |  |  |
| Australia | 1 | Spain | 1 |  |  |
| Brunei Darussalam | 1 |  |  |  |  |
| India | 1 |  |  |  |  |
| Japan | 1 |  |  |  |  |
| Maldives | 1 |  |  |  |  |
| Philippines | 1 |  |  |  |  |
| Sri Lanka | 1 |  |  |  |  |

**Table S2. General characterisation of the hospital participating in the HHEAs.** The first column includes 2017-2019 HHEA applications (HHSAF data: 2017-2019, ABHR data: 2012-2017), while the second includes 2021-2023 HHEA applications (HHSAF data: 2021-2023, ABHR data: 2015-2021).

| **Variable** | **Overall**  **(n=97)** | **2017-2019**  **(n=51)** | **2021-2023**  **(n=46)** | **p-value** |
| --- | --- | --- | --- | --- |
| Hospital type |  |  |  | **0.047** |
| General/University/Teaching | 50/85 (58.8) | 29/41 (70.7) | 21/44 (47.7) |  |
| Private | 35/85 (41.2) | 12/41 (29.3) | 23/44 (52.3) |  |
| Region |  |  |  | 0.305 |
| Asia-Pacific | 23/97 (23.7) | 12/51 (23.5) | 11/46 (23.9) |  |
| Europe | 14/97 (14.4) | 10/51 (19.6) | 4/46 (8.7) |  |
| Latin America | 60/97 (61.9) | 29/51 (56.9) | 31/46 (67.4) |  |

**Table S3. Output of the logistic regression models developed by choosing different cut-offs to discriminate high- versus low-ABHR consumption groups outside the central tertile of ABHR distribution (values in the central tertile are reported in Table 3 of the main manuscript).** For each cutoff *i* of ABHR consumption (dependent variable), hospitals were divided in 2 groups (high vs low) and the logistic regression was built as described in the Methods. The table shows the odds ratio (OR₁₀) of belonging to the high-consumption group for every 10-point increase in the HHSAF score (independent variable). Values reported in bold indicate cut-off values for which statistical significance is approached (*i* = 77-80).

| **Lower tertile (values < 30 ml/PD)** | | |  | **Upper tertile (values > 67 ml/PD)** | | |
| --- | --- | --- | --- | --- | --- | --- |
| **Cut-off (ml/PD)** | **OR [CI 95%]** | **p-value** |  | **Cut-off (ml/PD)** | **OR [CI 95%]** | **p-value** |
| ≤13 | 0.980 [0.755 - 1.271] | 0.876 |  | 68 | 1.049 [0.914 - 1.205] | 0.497 |
| 14 | 0.953 [0.745 - 1.219] | 0.701 |  | 69 | 1.039 [0.904 - 1.195] | 0.586 |
| 15 | 0.959 [0.767 - 1.199] | 0.714 |  | 70-74 | 1.090 [0.938 - 1.267] | 0.260 |
| 16 | 0.969 [0.789 - 1.189] | 0.761 |  | 75-76 | 1.142 [0.967 - 1.348] | 0.117 |
| 17 | 0.927 [0.752 - 1.141] | 0.474 |  | **77-80** | **1.204 [0.997 - 1.454]** | **0.054** |
| 18-20 | 0.924 [0.758 - 1.127] | 0.435 |  | 81 | 1.179 [0.979 - 1.420] | 0.083 |
| 21 | 0.986 [0.830 - 1.170] | 0.870 |  | 82 | 1.154 [0.960 - 1.388] | 0.126 |
| 22 | 1.040 [0.889 - 1.217] | 0.621 |  | 83-85 | 1.135 [0.944 - 1.364] | 0.179 |
| 23 | 1.055 [0.911 - 1.223] | 0.473 |  | 86-94 | 1.114 [0.927 - 1.339] | 0.250 |
| 24 | 1.024 [0.893 - 1.175] | 0.733 |  | 95-109 | 1.083 [0.903 - 1.299] | 0.389 |
| 25-28 | 1.087 [0.952 - 1.241] | 0.219 |  | 110-112 | 1.075 [0.892 - 1.297] | 0.448 |
| 29 | 1.090 [0.956 - 1.244] | 0.198 |  | 113-116 | 1.042 [0.865 - 1.255] | 0.668 |
|  |  |  |  | 117-118 | 1.015 [0.841 - 1.226] | 0.875 |
|  |  |  |  | ≥119 | 1.006 [0.825 - 1.225] | 0.956 |

**Additional File 3: Supplementary Figures**

**Figure S1 Distribution of scores for single MMIS elements of the HHSAF by region**. The violin plots represent scores obtained for System Change (a), Training and Education (b), Evaluation and Feedback (c), Reminders in Workplace (d) and Safety Climate (e). Each dot (statistical unit) represents a healthcare facility applying for the HHEA.


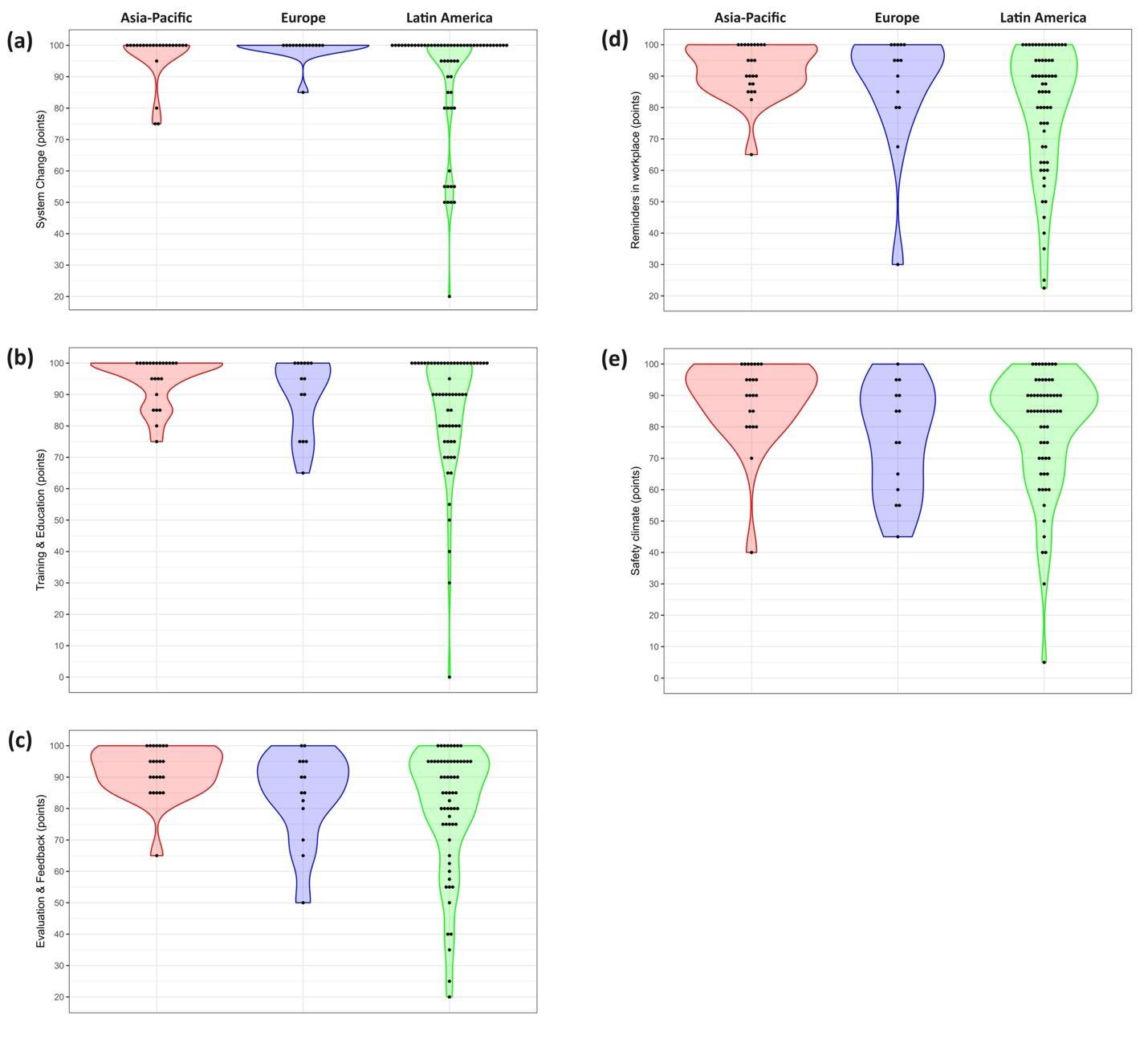


**Figure S2. Geographical representation of average HH performances in countries participating in the global Hand Hygiene Excellence Awards in the European region.** Each country is coloured according to the arithmetic mean of the HH compliance (a), total HHSAF scores (b), ABHR consumption (c) and ABHR consumption trend coefficients (d), of the respective hospitals participating in the HHEAs. This map considers 2012-2021 data for HH compliance and ABHR consumption, and 2017-2023 data for HHSAF scores. Cassini’s projection was used to build the maps.


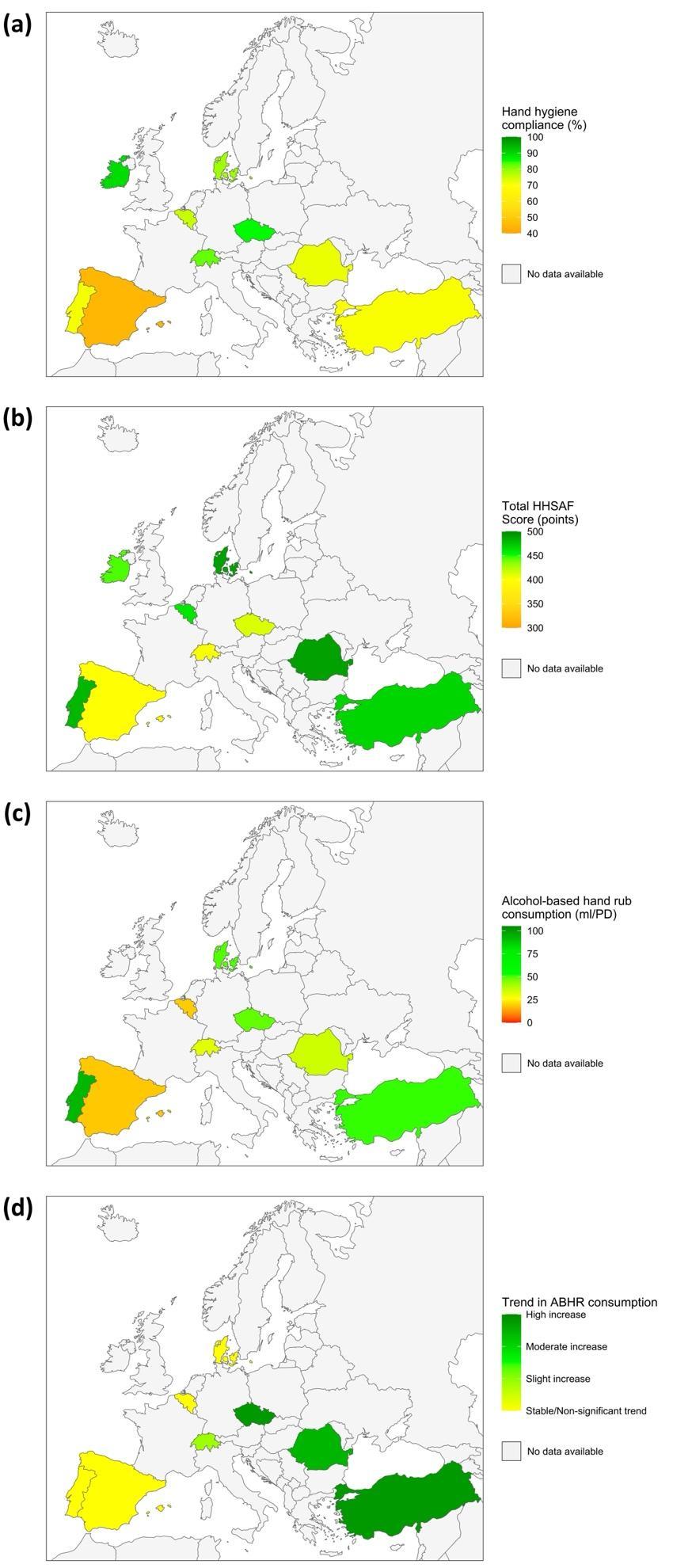


**Figure S3. Partitioning algorithm computed by the R package “partykit” (version 1.2-20) according to HHSAF score and ABHR consumption, with no group size constraint**. The best partition was obtained for an ABHR consumption threshold slightly above 76 ml/PD.


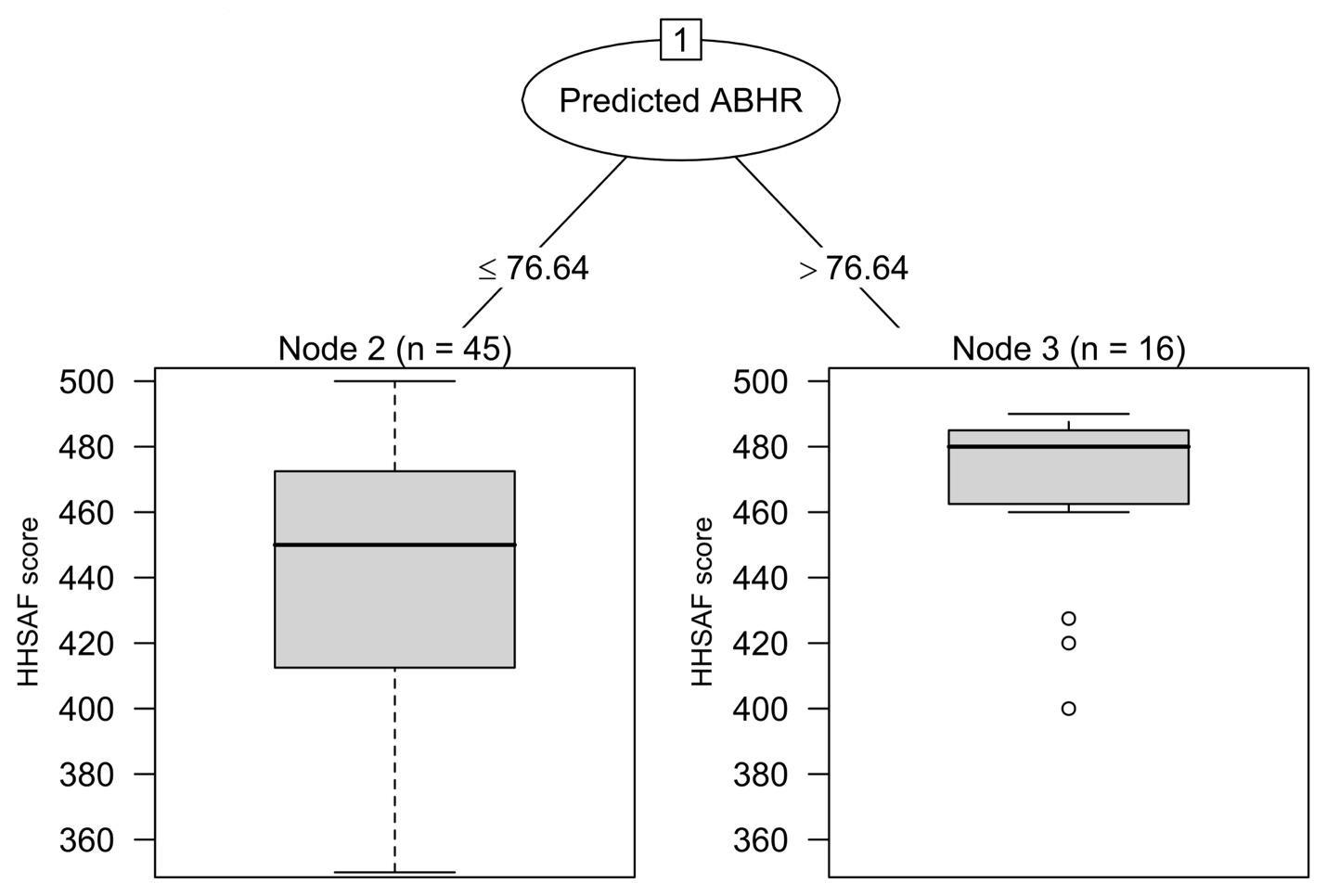

Supplement: Supplementary file 1 — Supplementary Material 1 [file 13756_2024_1399_MOESM1_ESM.docx]
